# Large outbreak of mumps virus genotype G among vaccinated students in Norway, 2015 to 2016

**DOI:** 10.2807/1560-7917.ES.2018.23.38.1700642

**Published:** 2018-09-20

**Authors:** Lamprini Veneti, Katrine Borgen, Kaja Sverdrup Borge, Kostas Danis, Margrethe Greve-Isdahl, Kirsten Konsmo, Gro Njølstad, Svein Arne Nordbø, Kari Stidal Øystese, Rikard Rykkvin, Eli Sagvik, Øystein Rolandsen Riise

**Affiliations:** 1 Division for Infection Control and Environmental Health, Norwegian Institute of Public Health, Oslo, Norway; 2 European Programme for Intervention Epidemiology Training (EPIET), European Centre for Disease Prevention and Control, (ECDC), Stockholm, Sweden; 3 Santé publique France, the French national public health agency (SpFrance), Saint-Maurice, France; 4 Department of Microbiology, Haukeland University Hospital, Bergen, Norway; 5 Department of Clinical and Molecular Medicine, Norwegian University of Science and Technology, Trondheim, Norway.; 6 Department of Medical Microbiology, St Olavs University Hospital, Trondheim, Norway; 7 Department of Infectious Disease Control, Municipality of Bergen, Bergen, Norway; 8 European Programme for Public Health Microbiology Training (EUPHEM), European Centre for Disease Prevention and Control, (ECDC), Stockholm, Sweden; 9 Department of Infectious Disease Control, Municipality of Trondheim, Trondheim, Norway

**Keywords:** mumps, outbreak, measles-mumps-rubella (MMR) vaccine, genotype G, students, Norway

## Abstract

From 6 September 2015–May 2016, a large mumps outbreak occurred among vaccinated students in Norway. A case was defined as a person presenting with a clinical mumps infection, notified between 1 September 2015 and 30 June 2016. Confirmed cases had positive laboratory confirmation and probable cases had an epidemiological link; PCR-positive specimens were genotyped. A total of 232 cases were notified (230 confirmed) with median age of 23 years (range 4–81) and 61% were male. Of 68 (30%) confirmed cases that were genotyped, 66 were genotype G and associated with the outbreak. Cases that had received two doses of the measles-mumps-rubella (MMR) vaccine had reduced risk of hospitalisation (adjusted relative risk (aRR): 0.14; 95%CI: 0.03–0.57), mumps-related orchitis (aRR: 0.21; 95% CI: 0.08–0.55) and severe outcome (aRR: 0.25; 95% CI: 0.10–0.62) compared with those unvaccinated. A third dose of the vaccine was offered to approximately 1,300 fully vaccinated close contacts and subsequently reported cases decreased. This large outbreak, occurring among predominately vaccinated students, suggests the current genotype A vaccine offers suboptimal protection against mumps genotype G. We recommend maintaining high vaccination coverage and offering the vaccine to all unvaccinated individuals.

## Introduction

Mumps is an acute infectious disease caused by a paramyxovirus. Mumps is usually spread by droplets, the incubation period is 16–18 days [1]. The infectious period starts 5 days before and up to 9 days after the onset of symptoms. Mumps patients usually present with parotitis, but at least one third of the infections are asymptomatic or present with non-specific symptoms such as fever and myalgia without parotitis. The disease is mostly self-limiting. Complications such as meningitis, pancreatitis, deafness and encephalitis can occur [1], with the most common being orchitis. During the 2009–2012 mumps outbreaks (in USA, Germany and Israel), orchitis occurred in up to 13% of the post-pubertal reported male cases [2-4].

Mumps is a notifiable disease and is reported via the Norwegian Surveillance system for Communicable Diseases (MSIS) by medical microbiological laboratories and clinicians to the Norwegian Institute of Public Health (NIPH). From 1975 to 1994 aggregated reporting forms were used, but since 1995, reports have been case-based (individual reports per case).

In 1983, mumps vaccination was introduced into the Norwegian childhood immunisation programme as part of the combined measles-mumps-rubella (MMR) vaccine. MMR vaccine has a median vaccine effectiveness estimated at 78% and 88% for one and two doses, respectively [5]. The vaccine is most protective in naïve populations and will have little or no effect if a person has already been exposed and infected by the virus. To date, only the genotype A strain, Jeryl Lynn (MMRII (SPMSD) and M-M-RVAXPro (MSD) and RIT 4385 (Priorix (GSK)) vaccine has been used in Norway. The first dose of the vaccine is offered to all children aged 15 months and the second to 11–12 year olds (6th grade). Since the introduction of vaccination, MMR vaccination coverage with at least two doses has exceeded 90% [6,7].

Before the introduction of the vaccine in Norway, mumps was very common with outbreaks occurring primarily in schools and military camps; following the introduction, the mean annual number of reported cases decreased from 52 cases during 1977–1983 to 16 cases during 1984-1999 with only sporadic cases occurring. During 2000–2014, mumps incidence remained low with a mean annual of cases of 15 (annual mean of 0.4 cases per 100,000 population). In 2006, a small local outbreak with 13 cases was reported.

### Alert

On 30 August 2015, St. Olavs University Hospital notified the municipal doctor of Trondheim about an unvaccinated international student in his early 20s, with recent travel history to Nepal, who had laboratory confirmed mumps infection. On 11 September, the municipal doctor was notified about a second laboratory confirmed mumps in an international student, also in his early 20s, with recent travel history to Italy who had two previous doses of MMR vaccine. These patients were studying at different universities, lived in different student dormitories and no epidemiological link was found through case investigation.

From 22 September to 1 October, 10 more students attending the same university as the second reported patient were diagnosed with mumps. None had an epidemiological link with either the first or second student (apart from studying at the same university as the latter). The number of patients notified with mumps continued to increase and the municipal doctor called NIPH for assistance to investigate the outbreak.

At the beginning of November, the mumps outbreak spread to the municipality of Bergen. The NIPH and the municipal doctors in Trondheim and Bergen began an outbreak investigation to estimate the extent of the outbreak, determine vaccination history of cases and provide recommendations for adequate control measures to prevent further spread of the disease.

We report on the investigation of this outbreak to describe its extent, the epidemiological characteristics and the control measures implemented to control it.

### Case definitions

MSIS uses the 2008 European Union (EU) mumps case definition and case classification [8]. A confirmed case was defined as a person with notified clinical mumps disease with laboratory confirmation (IgG seroconversion, IgM or PCR). A probable case was defined as a person with a notified clinical mumps and known epidemiological link to a case; a possible case was defined as a person with only clinical presentation of mumps. Cases infected abroad during the outbreak or with a non-outbreak strain were excluded. We included cases notified to MSIS until 30 June 2016 with symptoms onset from the 1 September 2015.

We defined severe cases as hospitalised mumps cases or those presenting with any mumps related complications (e.g. orchitis, meningitis).

Eligible MMR doses were defined as those administered at least 1 month before onset of any mumps symptoms. People younger than 11 years that received one dose of MMR vaccine, or those aged 11 years or older that received at least two doses of MMR vaccine were considered fully vaccinated. Those 11 years or older that received one dose of the vaccine were considered partially vaccinated. 

We considered close contacts as household members (e.g. sharing of student apartments), partners, or others who had close contacts (e.g. close friends) with cases.

### Laboratory investigation

Cases were laboratory-confirmed either through the detection of mumps virus in oral fluid specimens by reverse-transcriptase polymerase chain reaction (RT-PCR), viral culture or through serological confirmation by detection of anti-mumps specific IgM, IgG seroconversion, or significant increase in IgG titre. Isolated viruses from PCR positive cases were sequenced at the reference laboratory at NIPH to identify the genotype of the virus and examine if the cases were likely to be part of the same outbreak. This genotypic characterisation of the virus was based on the sequence of the most variable gene of the mumps genome, the SH gene, as previously described [9].

### Data collection

We linked MSIS epidemiological data with laboratory and vaccination data from the Norwegian vaccination registry (SYSVAK). When vaccination data was missing from SYSVAK we used information from MSIS. Since 1995, all vaccinations received within the Norwegian childhood immunisation programme have been recorded in SYSVAK [10]. Since 2011, health professionals in Norway are required to report vaccinations outside the childhood immunisation programme (travel vaccination, influenza etc.) to SYSVAK. Registration outside the childhood immunisation programme requires consent from the person vaccinated. Severe and suspected unexpected adverse events are reported to the NIPH.

Place of mumps infection was obtained from MSIS reports or from using the location of laboratories which tested the samples (e.g. St. Olavs University Hospital for Trondheim and Haukeland University Hospital for Bergen).

### Statistical analysis

We described cases in terms of vaccination status, mumps complications and severity of the disease. We calculated adjusted relative risks (aRRs) with 95% confidence intervals (CIs) using Poisson regression with a robust error variance to examine the associations between hospitalisation, mumps complications, overall severity of disease (outcomes) and eligible vaccine doses. We adjusted for age and sex, or only age when mumps orchitis (restricted analysis to male cases) was the outcome.

In addition, we also analysed only cases for whom vaccination dates were recorded in SYSVAK. We calculated the median number of years since the last MMR dose by subtracting the date of receipt of the last MMR dose from date of illness onset. We used Poisson regression to examine the association between severity of the disease and time since the last dose for male cases with at least two doses of the vaccine.

Statistical analysis was performed in Stata version 14 (Stata Corporation, College Station, Texas, US).

## Results

### Description of cases

**Figure 1:**
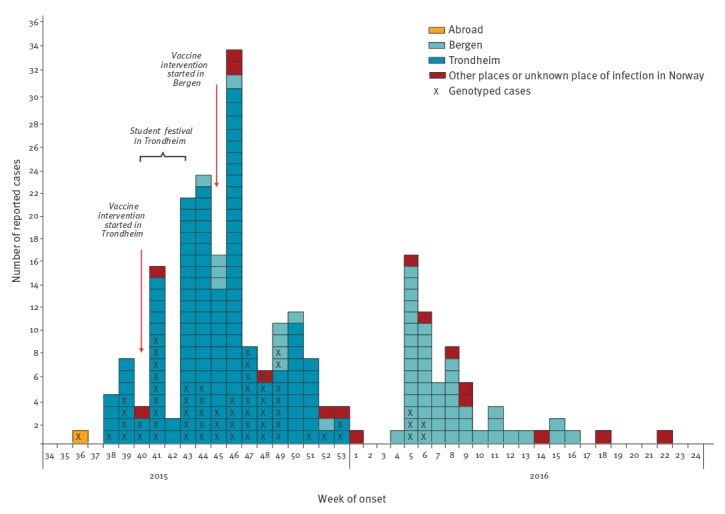
Number of reported mumps cases by week of onset and place of infection, Norway, September 2015–May 2016 (n = 232 cases)

Between September 2015 and May 2016, 232 mumps cases (230 confirmed and two probable) were reported (Figure 1). In 2015, the outbreak first occurred in the municipality of Trondheim and the number of cases peaked during the weeks 43–46 following one of the biggest student cultural festivals in the city of Trondheim. The festival took place from week 40–43 and around 90,000 tickets were sold for 200 events and concerts. The number of mumps cases in Trondheim decreased during the Christmas holidays, with the last case being notified to NIPH at the end of December. After the festival (week 44), the outbreak spread to other municipalities, but mainly affected students in Bergen, where the first cases reported were students that had attended the festival in Trondheim. In February 2016, the number of mumps cases increased in the municipality of Bergen, peaked during the weeks 5– 6 and then later decreased with the last case being notified to NIPH during week 16. At the beginning of February (week 5), the municipal doctor was informed about five students from Bergen who reported being sick, having symptoms compatible with acute mumps infection after having attending a student party at a ski resort at the end of January.

The median age of cases was 23 years and ranged from 4–81 years; 202 (87%) were 19–28 years old. Of all cases, 142 (61%) were male and 184 (79%) were Norwegians. The proportion of cases that were students exceeded 75%.

In total, there were 22 (9.5%) severe mumps cases reported, all male. No death was recorded. One (0.4%) case was diagnosed with meningitis, eight (3.5%) were hospitalised and 18 (13% among male cases) were diagnosed with orchitis.

### Laboratory results

During the outbreak, 69 specimens were received by the reference laboratory of NIPH in Oslo for genotyping; 68 were assigned to genotype G; one specimen could not be typed. Of the 68 isolates, 66 belonged to the outbreak. The 61 isolates were indistinguishable based on the sequence of the SH gene. Five isolates had up to three base differences in the sequenced genomic region compared with the rest, and were regarded as part of the outbreak based on sequence analysis combined with epidemiological information. Two samples (including the sample from the student with travel history to Nepal) had a large number of base differences compared with the rest and were therefore considered separate introductions/sporadic cases and unlikely to be a part of the outbreak.

The second reported student in Trondheim (with travel history to Italy) was defined as the primary case of the outbreak, since the case had an indistinguishable SH gene sequence compared with the rest of the outbreak cases. In contrast, the sequence from the sample from the student with travel history to Nepal who studied in Trondheim had a large number of base differences compared with the rest of the outbreak cases, indicating that this patient did not belong to the outbreak.

### Place of infection

**Figure 2:**
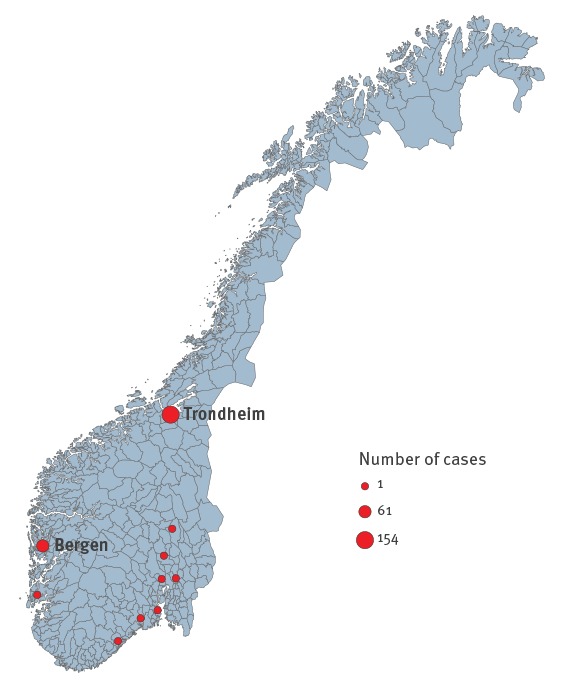
Place of infection of reported mumps cases in Norway, September 2015–May 2016 (n = 223)

Of 232 cases, the primary case was infected in Italy, 154 (66%) cases were infected in Trondheim, 61 (26%) in Bergen, eight (3.5%) in other municipalities in Norway and for eight (3.5%) cases the place of infection (in Norway) was unknown (Figure 2).

### Vaccination status and risk of severe disease

**Figure 3:**
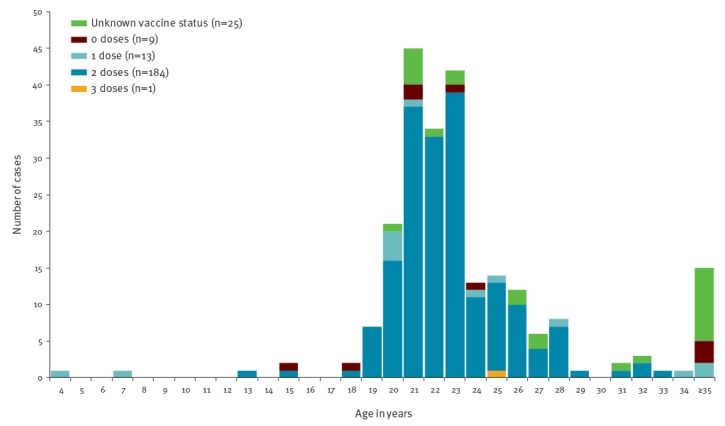
Distribution of mumps reported cases by age and measles-mumps-rubella (MMR) vaccination status, Norway, September 2015–May 2016 (n = 232 cases)

For 207 (89%) cases, the vaccination status was known and data on the number of vaccine doses received was obtained from either SYSVAK (n = 180; 78%) or MSIS (n = 23; 10%). Of those, one case had received three eligible MMR doses, 184 (89%) two doses, 13 (6.3%) one dose and 9 (4.4%) were unvaccinated (Figure 3). Approximately 41 (18%) reported cases were international students for whom vaccines were not always registered in SYSVAK of which, only 7/41 had their vaccination history registered in SYSVAK and 22/41 in MSIS.

When we restricted our analyses to the 180 cases that had vaccinations recorded in SYSVAK, one case had received three eligible MMR doses (received the third dose 12 years before the outbreak for unknown reasons), 162 (90%) has received two doses (fully vaccinated), 12 (6.7%) one dose and 5 (2.8%) were unvaccinated. Of the 162 cases with two doses, three had received a third dose of MMR during the outbreak but were reclassified as having received two eligible doses as their symptoms started within 2 weeks of receiving the third dose.

For the 175 cases that had received at least one eligible dose of MMR vaccination and for whom the vaccination dates were reported in SYSVAK, the median time from the last eligible dose to symptom onset was 10 years (inter-quartile range (IQR): 9–12 years). The median time interval between the two first doses was 11 years (IQR: 10.8–11.4) (Table 1).

**Table 1:** Time since last eligible dose of measles-mumps-rubella (MMR) vaccine to mumps symptom onset, Norway, September 2015–March 2016 (n = 175)

Characteristics of cases	MMR doses	Median (years)	Interquartile range (years)
Time since last eligible dose of MMR vaccine	1,2,3	10	9–12
	2 or 3	10	9–12
Time interval between two doses of MMR vaccine		11	10.8–11.4
Age	First	1	1–2
	Second	12	12–13
	Time of outbreak^a^	22	21–24

MMR: measles-mumps-rubella.

^a^Time of outbreak was 6 September 2015–May 2016.

All severe cases (n = 22/232) that were reported during the outbreak were male. Information regarding the number of MMR doses received was available for 20 (8.6%) of these cases; 8 (3.5%, 8/232) of which were hospitalised and 16 (11%, 16/142 male cases) had orchitis.

Cases that had received two doses of the vaccine had reduced risk of hospitalisation (aRR: 0.14; 95% CI: 0.03–0.57), mumps-related orchitis (aRR: 0.21; 95% CI: 0.08–0.55), and severe outcome (aRR: 0.25; 95% CI: 0.10–0.62) compared with those unvaccinated (Table 2).

**Table 2:** Association between mumps complications, hospitalisation, severe outcome and measles-mumps-rubella (MMR) vaccination status in mumps cases, Norway, September 2015–March 2016 (n = 207)

Complication or severe outcome	Doses of MMR vaccinations	Cases with complication	Total cases	%	Adjusted^a^ RR (95% CI)	p-value
Hospitalisation	0	2	9	22	Ref	Ref
	1	0	13	0	0.00 (0.00–0.00)	< 0.001
	2	5	184	3	0.14 (0.03–0.57)	0.006
	3	0	1	0	0.00 (0.00–0.03)	< 0.001
Orchitis^b^	0	4	7	57	Ref	Ref
	1	0	10	0	0.00 (0.00–0.00)	< 0.001
	2	12	103	12	0.21 (0.08–0.55)	0.001
Severe case^c^	0	4	9	44	Ref	Ref
	1	0	13	0	0.00 (0.00–0.00)	< 0.001
	2	15	184	8	0.25 (0.10–0.62)	0.003
	3	0	1	0	0.08 (0.01–0.62)	0.016

CI: confidence interval; MMR: measles-mumps-rubella; RR: relative risk.

^a^Adjusted for age (as continuous variable) and gender except for orchitis, which was adjusted only for age among males.

^b^Proportions and estimations for orchitis were calculated using data only from male cases with known vaccination status: 120 cases.

^c^A severe case was defined as a case that was hospitalised or presented with any mumps-related complications (e.g. orchitis, meningitis).

For the 93/142 male cases that had received two doses of MMR vaccination and for whom the dates of doses were reported (median age: 23; range: 13–33), the risk of orchitis increased by 16% (RR: 1.16; 95% CI: 1.01–1.34) for every additional year since the last MMR dose. In the model without time since last dose variable, the risk of orchitis increased by 18% (RR: 1.18; 95% CI: 1.03–1.36) for every additional year of age. However, when we included both variables together in the model (age and time since last dose), none of the aRRs were statistically significant (age aRR: 1.25; 95% CI: 0.94–1.66; time since last dose aRR: 0.95; 95% CI: 0.76–1.18). Additionally, the risk of hospitalisation or of severe outcome for those cases was not associated with increasing time since the last MMR dose (RR: 0.97; 95% CI: 0.71–1.32; RR: 1.13; 95% CI: 0.98–1.30; respectively).

### Implementation of control measures

After the first reports of confirmed mumps cases among students in Trondheim, the municipal public health authorities informed vulnerable groups (unvaccinated individuals and students) of the existence of the outbreak, the risk of transmission and control measures to increase their awareness. Risk communication channels included television, university newspapers, posters, local websites, university websites and e-mails to all students in all educational institutes in Trondheim. Further, the local public health authorities provided updated information to the local general practitioners, healthcare authorities and educational institutions during the outbreak.

The vaccine control measures in Trondheim started being implemented at the beginning of October (1–4 October, week 40) and in Bergen at the beginning of November (1–8 November, week 44–45). The recommended control measures included MMR vaccination of all unvaccinated or partially vaccinated students and general hygiene measures such as good hand hygiene, respiratory hygiene/cough etiquette and not sharing glasses/bottles and cutlery. People with mumps symptoms were advised to promptly seek medical advice for examination and stay at home for 7 days after symptom onset or until the laboratory results ruled out mumps. Physicians were advised to notify and take laboratory samples from all suspected mumps cases. Close contacts of cases were informed about the outbreak and that they had to be aware of the symptoms. Additionally, an extra dose of vaccine was offered to close contacts of cases, including a third dose to fully vaccinated contacts.

A third dose of vaccine was offered to 1,112 fully vaccinated close contacts of cases in Trondheim, (588 contacts during week 40–45 and 524 contacts during week 46–53) and 223 contacts in Bergen (during week 44/2015–16/2016). No serious adverse events were reported to the municipal doctors or to NIPH. During 18–19 November and 9–10 December (4 days) vaccination campaigns were organised at the university in Trondheim vaccinating almost 155 previously unvaccinated or partially vaccinated people (students or employees at the universities). During the outbreak period, 12 people were vaccinated in Bergen that were previously unvaccinated or partially vaccinated.

Local public health authorities were in charge of conducting case investigations and following up cases that were reported to be infected in other places apart from Trondheim and Bergen. Routine control measures were implemented for those cases including vaccination of all unvaccinated or partially vaccinated close contacts and general hygiene measures.

## Discussion

This was the first large mumps outbreak among vaccinated young adults in Norway, where MMR vaccine coverage has been constantly high (> 90%) in the last decade [6,7]. The exact cause of mumps outbreaks occurring among vaccinated populations remains unclear and while there are various possible reasons discussed but not confirmed [11]. We identified genotype G virus as the main variant type among our cases; similar genotype G mumps outbreaks have occurred the last decade in the US and Europe in the context of high two dose vaccination coverage [11-15]. The MMR vaccine strain used belongs to genotype A (Jeryl Lynn and RIT 4385), indicating that the mumps strains during this and other recent outbreaks were caused by different genotype than the vaccine strain. This may suggest a mismatch of the vaccine virus strain with the circulating outbreak strains. Genotype G might became more infectious than other mumps genotypes because of poorer cross-protection in individuals with waning antibody concentrations or specific immunopathogenic factors [13,15,16].

In our investigation, we observed that mumps cases vaccinated with two doses of the MMR vaccine had lower risk of complications and severe disease. This finding supports previous studies that suggest that the vaccine even if it does not fully protect against mumps infection, it still protects from severe mumps disease [17-20]. The protective effect of the MMR vaccination on disease severity is crucial in assessing the current and future mumps control strategies. In addition, it was mostly vaccinated young adults who had their last MMR dose 10 years before that were affected, which could point towards waning immunity as indicated from other studies [21-23].

The percentage of male cases (13%, 18/142) with orchitis identified in our investigation were similar to the proportion reported during other mumps outbreaks [17,18]. We observed that for male cases with at least two doses of the vaccine (95% of them were 20–28 years old), the risk of orchitis increased for every additional year in time since the last MMR dose. Even though there might be a correlation of time since the last dose with age, this could still suggest that waning immunity may result in more frequent mumps orchitis. Even though there are studies reporting that mumps immunity wanes with increasing time since last vaccination [22,23], we could not find any published study that examined the association between time since last dose and mumps severity (e.g. hospitalisation and/or mumps complications).

The United States Centres for Disease Control criteria for considering a third dose of the vaccine as a control measure, that supported NIPH’s decision, include outbreaks among populations with high two dose vaccination coverage (> 90%), intense exposure settings (such as army camps, schools etc.), high attack rates (> 5 cases per 1,000 population), and evidence of ongoing transmission for at least 2 weeks in the target population (population with high attack rates) [5]. In our outbreak, all criteria could be applied apart from the criterion of high attack rates. We could not estimate the attack rate, as we did not know the exact number of students present in the different affected universities and could not estimate the exact number of students among cases.

The NIPH recommended a third dose of the vaccine only to close contacts of cases and not to all students in the affected universities. This decision was taken considering the limited evidence and the cost of such intervention. To our knowledge, this is the first outbreak that a third dose of the MMR vaccine was provided only to close contacts of cases. If the outbreak had continued, one of the strategies could have been a third dose of the MMR vaccine to all students who had two previous doses, although the impact of such a strategy is not well documented.

Among the approximately 1,300 close contacts of cases who received the third dose of the vaccine, only three became cases during the outbreak period. These cases might have been exposed to mumps virus before their vaccine-induced immunity was boosted, as all three cases had an onset of symptoms within 2 weeks of receiving the third dose, and thus the dose was not eligible. This might suggest that administering a third dose of the vaccine to close contacts of cases may have contributed to the prevention of new cases during this outbreak in a close setting. No serious adverse events were reported to people who received the third dose of the vaccine.

Even though the vaccine effectiveness of a third dose of MMR vaccine has not been established, there is some evidence that it induces an immune response with a couple of outbreaks showing significantly declining attack rates after a third dose intervention [24,25]. However, there has also been an outbreak in which the attack rates did not decline significantly after a third dose intervention [26]. We did not calculate the attack rates, but we noticed that after the vaccine control measures started being implemented in Trondheim following the festival, the number of reported cases decreased.

The outbreak first occurred among students following the biggest student cultural festival in Trondheim; students travelled from different places of Norway to attend this festival. The close contact of students during crowded events in not well ventilated areas can facilitate the spread of mumps. Crowding at universities, sharing student dormitories and high contact rates have been previously reported to contribute to the spread of mumps outbreaks [11].

During this outbreak, local public health authorities used all available means for risk communication to increase awareness among the vulnerable groups. Although the number of cases decreased following those interventions and the outbreak stopped, we could not determine whether this decrease was attributable to (i) the natural course of the outbreak, (ii) the third dose intervention combined with the catch-up vaccination of unvaccinated students or (iii) the reduced opportunities of close contact since the festival was over and by following the recommendations (hygiene measures, people with mumps symptoms should stay home). Moreover, the outbreak in Trondheim ended during Christmas holidays, most likely a result of students staying away from high-density settings; specific recommendations were also given to mumps cases before going back home for Christmas in order to prevent further spread of the disease.

Genotyping assisted NIPH to monitor the outbreak, identify the primary case and confirm that one single outbreak strain circulated in Norway. At the beginning of the outbreak, an unvaccinated student in Trondheim with travel history in Nepal was considered to be the primary case, but sequencing confirmed that he did not belong to this outbreak. No epidemiological or laboratory link was found among the cases reported during the outbreak with this student; therefore we assume that he did not lead to a separate outbreak.

### Limitations

Our study had several limitations. First, we did not estimate the third dose vaccine effectiveness since we did not have data available on the number of students in the two affected cities, the exact number of students among cases and the vaccination coverage of the affected population. Second, there might have been (i) over-ascertainment of severe cases compared with less severe cases, with more severe cases being more likely to visit a clinician and (ii) under-ascertainment of cases and complications as several cases may have not visited a clinician or complications may occurred after the first visit. Although clinicians reported complications to MSIS as soon as a case was identified, some complications could have occurred later i.e. after the first visit of the patient. We did not routinely ask for follow-up data on cases reported to MSIS, although this was done for a few cases. We cannot evaluate how exactly these have influenced the number of severe cases reported but we assume that the same issue existed in other mumps outbreaks and our results are comparable. Moreover, the poor sensitivity of parotitis IgM tests in vaccinated patients [27,28] may also have contributed to an underestimation of cases.

## Conclusion and recommendations

This large mumps outbreak in Norway occurred mainly among vaccinated students and suggests the current genotype A vaccine provides suboptimal protection against genotype G mumps outbreaks; the exact cause of mumps outbreaks among vaccinated students remains unclear, but the outbreak may have been larger if more of the population were unvaccinated. MMR vaccination was associated with less severe disease among mumps cases. We recommend maintaining high vaccination coverage of two doses of MMR vaccine among children and students and offer the vaccine to all unimmunised individuals. Rapid recognition of mumps by healthcare providers also remains essential. In addition, vaccine effectiveness studies in outbreak settings are needed in order to estimate the effectiveness of administering a third dose of the vaccine, the results of which would better inform decision-making during outbreak responses and instigate potential changes to current recommendations. Future studies in outbreak settings should also evaluate the impact of the different outbreak control measures including behavioural factors, hygiene measures and vaccine control measures.
